# Clinical Significance and Prognostic Impact of Subcutaneous or Intrastrap Muscular Recurrence of Papillary Thyroid Carcinoma

**DOI:** 10.1155/2012/819797

**Published:** 2011-08-08

**Authors:** Yasuhiro Ito, Mitsuyoshi Hirokawa, Takuya Higashiyama, Yuuki Takamura, Kaoru Kobayashi, Akihiro Miya, Akira Miyauchi

**Affiliations:** ^1^Department of Surgery, Kuma Hospital, 8-2-35, Shimoyamate-dori, Chuo-ku, Kobe 650-0011, Japan; ^2^Department of Pathology, Kuma Hospital, 8-2-35, Shimoyamate-dori, Chuo-ku, Kobe 650-0011, Japan

## Abstract

Subcutaneous or intrastrap muscular (SIM) recurrence is rare in papillary thyroid carcinoma (PTC) patients, and its clinical significance remains unclear. We analyzed 29 patients with PTC who showed SIM recurrence in order to elucidate this issue. The incidence of patient age 55 years or older at initial surgery, extrathyroid extension, and clinically detected lymph node metastasis was 83%, 35%, and 46%, respectively. After surgical dissection, 17% of patients showed repeated SIM recurrence. Distant recurrence was detected in 45% of patients and was more likely to occur in patients with high-risk clinicopathological features. In all but one patient in this series, distant recurrence was detected at the same time or after the detection of SIM recurrence. Three patients died of PTC, but none of these patients died of the development of recurrent SIM lesions. These findings suggest that although SIM recurrence is a rare event and is not fatal, it is a predictor of distant recurrence especially in patients with high-risk clinicopathological features. Careful followup is recommended for such patients.

## 1. Introduction

Papillary thyroid carcinoma (PTC) is usually an indolent disease showing an excellent prognosis if competently resected. However, it is also well known that PTC can recur to various organs after surgery. The organ to which PTC will most likely recur is the regional lymph node [1], and such recurrence is not immediately life threatening with a few exceptions. However, PTC can also recur in distant organs such as the lung, bone, and brain. Although distant recurrence is less frequent, it can be fatal if recurrent lesions are progressive and refractory to radioactive iodine (RAI) therapy. 

 Subcutaneous or intrastrap muscular (SIM) recurrence is a rare site for PTC recurrence. The most common cause of SIM recurrence is needle tract implantation during fine needle aspiration biopsy (FNAB) for the diagnosis of PTC. We previously showed that needle tract implantation occurred in 0.14% of patients who underwent FNAB for PTC [2]. Other potential etiologies of SIM recurrence are attachment and proliferation of carcinoma cells transported in the bloodstream to subcutaneous tissue or intrastrap muscle, lymphatic metastasis to subcutaneous lymph node, and intraoperative dissemination of carcinoma cells. 

 Little is known about the clinical significance of SIM recurrence possibly because of its rarity. To date, we encountered 29 patients with PTC who showed SIM recurrence, and in this study, we investigated the impact of this event on the clinical course of PTC patients.

## 2. Patients and Methods

Between 1987 and 2008, 8976 patients underwent initial surgery for PTC. These patients underwent fine needle aspiration biopsy (FNAB) before surgery and were diagnosed as having PTC. After surgery, surveillance for local recurrence was performed at least once per year by ultrasonography. To date, 29 patients (0.29%) have shown SIM recurrence during followup. All these lesions were diagnosed as PTC recurrence on FNAB or thyroglobulin measurement of the washout of needles [3]. Similarly, 3 patients who underwent initial surgery at other hospitals, and then received postoperative followup in our hospital showed SIM recurrence. These 29 patients were enrolled in this study. They consisted of 24 females and 5 males, and patient ages at the initial surgery ranged from 30 to 79 years (average 61 years). The extent of thyroidectomy at initial surgery was total thyroidectomy in 14 patients and limited thyroidectomy in the remaining 15 patients. The extent of lymph node dissection included both the central and lateral compartments in 24 patients and only central node dissection in 2 patients. Lymph node dissection was not performed in one patient. The extent of node dissection was unknown in the remaining 2 patients. None of these patients showed distant metastasis at initial surgery. Patients who showed other accompanying thyroid malignancies in the primary lesions or in lymph node metastases such as anaplastic carcinoma, poorly differentiated carcinoma on WHO classification or Turin proposal [4, 5], follicular carcinoma, medullary carcinoma, and malignant lymphoma were excluded from the series. Patients whose regional lymph node metastasis directly invaded the subcutaneous tissue and whose other recurrent lesions were diagnosed as anaplastic carcinoma before the detection of SIM recurrence were also excluded. To date, 28 of 29 patients underwent surgical dissection of recurrent lesions that were diagnosed as PTC on pathological examination. The intervals between initial surgery and appearance of SIM recurrence ranged from 10 to 172 months, and the average interval was 79 months. Distant recurrence was evaluated on chest CT scan, roentgenography, or PET-CT scan. The entire follow-up period from initial surgery ranged from 28 to 234 months (average 137 months). 

## 3. Results

To date, 29 patients with PTC showed SIM recurrence. Backgrounds and preoperative clinical features of these patients at initial surgery are summarized in Table 1. Twenty-four patients (83%) were aged 55 years or older at the initial surgery, and 20 (76%) had primary lesions larger than 2 cm. Twelve patients (46%) were classified as N1a or N1b on the UICC TNM classification [6], because they showed lymph node metastasis detectable on preoperative imaging studies, and 4 (15%) had node metastasis measuring 3 cm or larger. Ten patients (38%) were classified as Stage IVA [6], while only 4 (15%) were classified as Stage I. 

Table 2 indicates intraoperative and pathological findings at initial surgery for these 29 patients. Extrathyroid extension corresponding to T4 on TNM classification [6] was detected in 9 patients (35%). Similarly, node metastasis of 3 patients (12%) extended to adjacent organs requiring at least partial resection of these organs. On pathological examination, lymph node metastasis was detected in 24 patients (92%), and 20 (77%) were graded as pStage IVA.

 Clinical courses for these patients are demonstrated in Table 3. All but one patient, who rejected further therapy, underwent surgery for SIM recurrence, and all were pathologically diagnosed as having PTC. The remaining one was diagnosed as having PTC on cytology. Subcutaneous or intrastrap muscular tissue was the first organ showing PTC recurrence in 19 patients (66%), and 12 (41%) have shown no further recurrence to date. However, 5 patients (17%) developed repeated SIM recurrence. Four of these 5 patients showed recurrence twice, and the remaining one showed recurrence three times. Two patients underwent external beam radiotherapy to the neck after surgical dissection of SIM recurrence. One of these patients has shown no further recurrence to the neck to date, but another showed further SIM recurrence even after external beam radiotherapy. Seventeen patients (59%), including the remaining 7 (19 minus 12), showed recurrence to other organs. Fifteen patients (52%) showed local recurrence, and 13 (45%) showed distant recurrence. Eleven patients showed both local and distant recurrences. Local organs to which PTC recurred were the lymph node in 13 patients and the remnant thyroid in 2 patients. Distant organs showing recurrence were the lung in 11 patients, bone in 2 patients, and brain in 1 patient. The patient showing recurrence to the brain also had recurrence to the lung.

 Of 13 patients showing distant recurrence, only 1 showed SIM recurrence after distant recurrence. The interval between two recurrences was 46 months. Distant recurrence and SIM recurrence were simultaneously observed in 4 patients. The remaining 8 showed distant recurrence after the detection of SIM recurrence, and the intervals between these recurrences ranged from 10 to 77 months. 

 To date, 3 of 13 patients who showed distant recurrences have died of PTC 17, 52, and 69 months after the detection of SIM recurrence, respectively (Table 3). Two patients showed preoperatively detectable node metastasis, and the primary lesions in two patients showed extrathyroid extension. None of the patients in our series died of uncontrollable growth, including anaplastic transformation, of SIM recurrence.

Table 4 summarizes the relationship between distant recurrence and clinicopathological features of 29 patients with SIM recurrence. All patients who showed distant recurrence were aged 55 years or older. Patients showing clinical node metastasis were significantly more likely to show distant recurrence than those without clinical node metastasis. Especially, all 3 patients who had extranodal tumor extension showed distant recurrence after the detection of SIM recurrence. Although there was no significant difference, patients with extrathyroid extension tended to show distant recurrence more frequently than those without extrathyroid extension.

## 4. Discussion

In this study, we reviewed the records of patients with SIM recurrence in order to elucidate the clinical significance of this rare event. There were some notable characteristics of patients who showed SIM recurrence. We previously showed that age 55 years or older, clinical node metastasis (especially large node metastasis), extrathyroid extension, and extranodal tumor extension were important signs of biological aggressiveness of PTC [1, 7]. In the series of patients with SIM recurrence, the incidences of patients aged 55 years or older, clinical node metastasis, node metastasis larger than 3 cm, extrathyroid extension, and extranodal tumor extension were 83%, 46%, 15%, 35% and 12%, respectively. We analyzed 5911 patients with PTC who underwent surgery between 1987 and 2006, and these incidences in this series were much lower at 40%, 20%, 3%, 13%, and 2%, respectively [7]. It is therefore suggested that SIM recurrence is likely to occur in PTC patients displaying high-risk features.

 We have to note that 13 patients in our series also had distant recurrence, accounting for as much as 45%. All patients except one showed distant recurrence after or at the same time as detection of SIM recurrence. These findings strongly suggest that SIM recurrence is a strong predictor of distant recurrence. This is possibly because PTC showing such an unusual lesion, whatever its mechanism, shows high metastatic activity and proliferating activity in metastatic lesions. It is also noteworthy that 66% of patients showed SIM recurrence as the initial sign of recurrence. Therefore, when we encounter SIM recurrence, an immediate search for other metastases is recommended. Even when no other recurrences could be detected, these patients should thereafter undergo more careful followup than previously. We also showed that patients with high-risk features such as advanced age, extrathyroid extension, and clinical node metastasis were more likely to show distant recurrence. Therefore, we must prepare for a high incidence of distant recurrence, especially in patients with high-risk features, when SIM recurrence is detected.

 To date, 3 patients have died of PTC but none of these died of SIM recurrence, indicating that SIM recurrence is generally not fatal at least in our series. This may be because all patients except one underwent surgery immediately after the detection of SIM recurrence. However, 17% of patients showed repeated SIM recurrence. We performed external beam radiotherapy to the neck for two patients. One patient did not develop further recurrence thereafter, but the other showed further recurrence. Therefore, it remains unknown whether external beam radiotherapy is effective for SIM recurrence, but it may be a therapy option after surgical dissection. 

 For patients whose SIM recurrence is detected as an initial recurrence of PTC and other recurrent lesions were simultaneously detected, surgery should be performed not only for SIM recurrence but also for other lesions. Furthermore, if patients underwent limited thyroidectomy at the initial surgery, completion total thyroidectomy should be performed because such patients will show high incidences of distant recurrence requiring RAI therapy, and followup of thyroglobulin level will not be necessary.

 In summary, SIM recurrence in PTC patients is a rare event and is not fatal. However, SIM recurrence is a predictor of distant recurrence especially for patients with high-risk factors, regardless of the mechanism underlying SIM recurrence. Careful investigation of other recurrences at the time SIM recurrence is detected and careful followup after surgical dissection are highly necessary for such patients.

## Figures and Tables

**Table 1:** Backgrounds and clinical features at initial surgery that were preoperatively evaluated for 29 PTC patients showing SIM recurrence.

Gender		
Male	5 (17%)	
Female	24 (832)	
Age (yrs)		
≥55	24 (83%)	
<55	5 (17%)	
Family history of PTC		
Yes	0	
No	29 (100%)	
Size of primary lesions (cm)		
>4	3 (11%)	
2.1−4	17 (65%)	
1.1−2	5 (20%)	
≤1	1 (4%)	(3 unknown)
Clinical node metastasis (*N*)		
N1b	11 (42%)	
N1a	1 (4%)	
N0	14 (54%)	(3 unknown)
Lymph node metastasis ≥3 cm		
Yes	4 (15%)	
No	22 (85%)	(3 unknown)
Stage		
IVA	10 (38%)	
III	1 (4%)	
II	11 (42%)	
I	4 (15%)	(3 unknown)
Multiplicity on imaging studies		
Multiple	6 (23%)	
Solitary	20 (77%)	(3 unknown)

**Table 2:** Intraoperative and pathological findings at initial surgery for 29 PTC patients showing SIM recurrence.

Extrathyroid extension		
Yes	9 (35%)	
No	17 (65%)	(3 unknown)
Extranodal tumor extension		
Yes	3 (12%)	
No	23 (88%)	(3 unknown)
Pathological node metastasis		
Yes	24 (92%)	
No	2 (8%)	(3 unknown)
Pathological multiplicity		
Yes	10 (38%)	
No	16 (62%)	(3 unknown)
p Stage		
IVA	20 (77%)	
III	0	
II	2 (8%)	
I	4 (15%)	(3 unknown)

**Table 3:** Clinical courses for 29 PTC patients showing SIM recurrence.

*Was this the initial recurrence?	
Yes No	19 (66%) 10 (34%)
Repeated SIM recurrence	
Yes No	5 (17%) 24 (83%)
Recurrence at other sites	
Yes No	17 (59%) 12 (41%)
Other local recurrences	
Yes No	15 (52%) 14 (48%)
Distant recurrences	
Yes No	13 (45%) 16 (55%)
Clinical outcomes	
Death of PTC Alive with PTC Alive without PTC	3 (10%) 10 (34%) 16 (56%)

*Including patients whose other recurrenceswere detected at the same time.

**Table 4:** Relationship between clinicopathological features at initial surgery and distant recurrence.

Distant recurrence	Yes (*n* = 13)	No (*n* = 16)	*P* values	
Gender				
Male	1 (20%)	4 (80%)		
female	12 (50%)	12 (50%)	0.2198	
Age (yrs)				
≥55	13 (54%)	11 (46%)		
<55	0 (0%)	5 (100%)	0.0267	
Tumor size (cm)	3.0 ± 1.2	2.8 ± 1.2	0.6805	(3 unknown)
Extrathyroid extension				
Yes	5 (56%)	4 (44%)		
No	6 (35%)	11 (65%)	0.3198	(3 unknown)
Clinical node metastasis (*N*)				
Yes	8 (73%)	3 (27%)		
No	3 (21%)	11 (79%)	0.0199	(3 unknown)
Repeated SIM recurrence				
Yes	2 (40%)	3 (60%)		
No	11 (46%)	13 (54%)	0.8114	
